# Clinical value of the low-grade inflammation score in aneurysmal subarachnoid hemorrhage

**DOI:** 10.1186/s12883-023-03490-2

**Published:** 2023-12-11

**Authors:** Ling wang, Ling Chen, Yang Jin, Xiangyang Cao, Liujun Xue, Qiantao Cheng

**Affiliations:** 1 Department of Neurology, Changhai Hospital, Second Military Medical University, Shanghai, China; 2 Department of Neurology, The First Affiliated Hospital of Anhui Medical University, Hefei, China; 3 Department of Neurology, The Affiliated Huai’an Hospital of Xuzhou Medical University, Huaian, Jiangsu China; 4 Department of Neurology, The Affiliated Huai’an No.1 People’s Hospital of Nanjing Medical University, Huaian, Jiangsu China; 5 Department of Neurology, Huai’ an 82 hospital, Huaian, Jiangsu China

**Keywords:** Aneurysmal subarachnoid Hemorrhage, Low-grade inflammatory score, Symptomatic cerebral vasospasm, Poor functional outcome

## Abstract

**Background and purpose:**

Multiple inflammatory biomarkers have been shown to predict symptomatic cerebral vasospasm (SCVS) and poor functional outcome in patients with aneurysmal subarachnoid hemorrhage. However, the impact of the low-grade inflammation (LGI) score, which can reflect the synergistic effects of five individual inflammatory biomarkers on SCVS and poor functional outcome on aneurysmal subarachnoid hemorrhage (aSAH), has not yet been well established. The aim of this study was to evaluate the impact of the LGI score on SCVS and poor functional outcome in aSAH patients.

**Methods:**

The LGI score was calculated as the sum of 10 quantiles of each individual inflammatory biomarker. The association of the LGI score with the risk of SCVS and poor functional outcome was analyzed with multivariate logistical regression.

**Results:**

A total of 270 eligible aSAH patients were included in this study: 74 (27.4%) had SCVS, and 79 (29.3%) had poor functional outcomes. After adjusting for confounders, a higher LGI score was revealed to independently predict SCVS (OR, 1.083; 95% CI, 1.011–1.161; *P* = 0.024) and poor functional outcome (OR, 1.132; 95% CI, 1.023–1.252; *P* = 0.016), and the second and third tertile group had higher risk of SCVS than lowest tertile group (OR, 2.826; 95% CI, 1.090–7.327; *P* = 0.033) (OR, 3.243; 95% CI, 1.258–8.358; *P* = 0.015). The receiver operating characteristic (ROC) curve uncovered the ability of the LGI score to distinguish patients with and without SCVS (area under the curve [AUC] = 0.746; 95% CI, 0.690–0.797; *P* < 0.001) and poor functional outcomes (area under the curve [AUC] = 0.799; 95% CI, 0.746–0.845; *P* < 0.001), the predictive value of LGI on SCVS and poor functional outcome is superior than PLT, NLR and WBC, but there was no statistical difference between LGI and CRP for predicting SCVS (*P* = 0.567) and poor functional outcome (*P* = 0.171).

**Conclusions:**

A higher LGI which represents severe low grade inflammation status is associated with SCVS and poor functional outcome at 3 months after aSAH.

## Introduction

Aneurysmal subarachnoid hemorrhage (aSAH) is the most common type of spontaneous subarachnoid hemorrhage (SAH) [1] and is a life-threatening neurological emergency in clinical practice. Patients with aSAH often experience a sudden onset and a rapid progression of symptoms and have high rates of mortality and permanent disability [2]. Although the rapid development of neurosurgical and neurointensive care techniques has improved the patient prognosis, those with severe systemic inflammatory responses still have high rates of mortality and disability [3]. Systemic inflammatory response syndrome occurs in up to 87% of patients after aSAH [4]; although the specific pathophysiological mechanism is not clear, it has been strongly associated with cerebral vasospasm and delayed cerebral infarction (DCI) [3]. Therefore, several peripheral inflammatory biomarkers are widely used in the clinical risk assessment of patients with aSAH. C-reactive protein (CRP), white blood cells (WBCs) and the neutrophil-to-lymphocyte ratio (NLR) were confirmed to be reliable predictors of various complications and poor functional outcomes after aSAH [5, 6]. However, most of the studies used single biomarker approaches or the ratio of two indicators rather than considering a panel of combined biomarkers.

Low-grade inflammation (LGI) is recognized as a risk factor for several chronic diseases, including cardiovascular disease, cancer and neurodegenerative disease [7–9]. The low-grade inflammation score (LGIS) has been used previously to evaluate the possible synergistic effects of each inflammatory biomarker (CRP, WBC, platelet count, and NLR) [10]. This novel index can independently predict total mortality in the healthy adult general population and cardiovascular mortality in patients with cardiovascular diseases [11, 12]. In addition, an elevated LGIS is associated with a higher risk of stroke recurrence [13]. In aSAH patients, inflammatory biomarkers change simultaneously; therefore, considering the synergistic effects might better illustrate the level of inflammation. To date, few studies have confirmed the prognostic value of LGIS in aSAH patients; therefore, this study aimed to use this predefined LGIS to investigate its relationship with symptomatic cerebral vasospasm (SCVS) and poor functional outcome in aSAH patients.

## Methods

### Study populations

In this retrospective study, data were collected from consecutive patients diagnosed with aSAH at multiple research centers, including the Department of Neurology, The First Affiliated Hospital of Anhui Medical University, Huai’an Hospital and Huai’an No. 1 People’s Hospital, from September 2018 to June 2021. The inclusion criteria were as follows: (1) diagnosed with SAH by computed tomography (CT), aneurysms detected by computed tomography angiography (CTA) and digital subtraction angiography (DSA); (2) admission 48 h after onset and laboratory examination; and (3) age of 18 years or older. The exclusion criteria were as follows: (1) nonaneurysmal SAH, such as trauma, vasculitis, and arteriovenous malformation rupture; (2) severe hepatic or renal disease, hematological disease, malignant tumor, autoimmune disease and immunosuppressive therapy; and (3) incomplete clinical data. All patients or their legal representatives signed informed consent, and the protocol was approved by the Ethics Committee of The First Affiliated Hospital of Anhui Medical University.

### Data collection

Patient demographics, vascular risk factors (such as hypertension, diabetes mellitus, history of smoking), surgical approaches and aneurysm location were all collected and evaluated. The severity of clinical presentation at admission was assessed by the World Federation of Neurological Societies (WFNS) grade and Hunt-Hess classification [14]. The modified CT Fisher grade was used to assess SAH on CT scans [15]. Functional outcome was defined according to the modified Rankin Scale (mRS) at discharge, and an mRS score of 3–6 suggested a poor functional outcome. Blood samples were monitored shortly after admission; the white blood cell count (WBC), neutrophil count, lymphocyte count and CRP were all collected; and the NLR was defined as the neutrophil count divided by lymphocyte count.

### Assessment of LGIS

LGIS was introduced to evaluate the synergistic effects of inflammatory biomarkers (CRP, WBC, PLT and NLR). The value of each biomarker was divided into 10 quantiles; the highest deciles (7 to 10) had a score that increased from 1 to 4, while the lowest deciles (1 to 4) were negatively scored from − 4 to -1 and deciles 5 or 6 received zero points. Then, the values of the four biomarkers were summed to obtain the LGIS, and this total score represented the intensity of low-grade inflammation, ranging from − 16 to 16 [10]. Patients were stratified into three groups (T1-T3) according to the LGI value; the higher the LGI index, the more severe the low-grade inflammation was considered to be.

### Definition of symptomatic cerebral vasospasm

Symptomatic cerebral vasospasm was defined as the development of new focal neurological signs and deterioration in the level of consciousness, or both. The cause of deterioration was considered to be cerebral ischemia attributable to vasospasm after excluding other possible causes (rebleeding, hydrocephalus, seizures, metabolic derangement, infection, excessive sedation, hypotension, hypoxia, fever, heart failure, and cerebral edema) [16]. In our study, SCVS was evaluated by two certified neurologists blind to clinical data.

### Statistical analysis

Statistical analysis was conducted using the Statistical Package for the Social Science, version 26.0 (SPSS Inc., Chicago, IL) and Medcalc 19. Continuous variables are described herein as the mean (standard deviation) and median (interquartile ranges, IQRs), and categorical variables are expressed as numbers (percentages). Differences in baseline characteristics were assessed by the Chi-square or Fisher exact test for categorical variables and by the *t* test, Mann‒Whitney U test, one way analysis of variance, and Kruskal‒Wallis test as appropriate.

The collinearity between candidate variables was examined using variance inflation factors before developing the multivariable binary logistical regression model, which was used to analyze the predictive value of the LGI score for SVCS and poor outcome. We used the lowest tertile as the reference category. The covariates entered in the multivariate logistical regression to evaluate the association of LGI score and SCVD were age, gender, CT Fisher grade, WFNS grade and albumin, we further adjusted SCVS and hydrocephalus in the multivariate logistical regression for predicting poor functional outcome. Two-tailed *P* values of < 0.05 were considered statistically significant. Receiver operating characteristic (ROC) curve analysis was performed to examine the discrimination of the LGI score and each individual biomarkers of the score. Pairwise comparison was performed using the Delong’s test.

## Results

### Baseline characteristics

A total of 270 eligible aSAH patients were included in this study: 74 (27.4%) had SCVS, and 79 (29.3%) had poor functional outcomes. Table 1 shows the baseline clinical characteristics of patients stratified by the tertile of the LGI score. Patients in the higher LGI score group were older (59 vs. 58 vs. 62; *P* = 0.027) and more likely to have a high Hunt-Hess grade (2.5% vs. 14.3% vs. 36.6%; *P* < 0.001) and WFNS grade (12.2% vs. 37.8%. vs. 56.2%; *P* < 0.001); a higher CT Fisher grade (12.7% vs. 29.6% vs. 57.0%; *P* < 0.001); and a higher incidence of poor outcomes and SCVS (8.9% vs. 21.4% vs. 54.8%; *P* < 0.001) (12.7% vs. 23.5% vs. 44.1%; *P* < 0.001). Compared with the patients in the first tertile group, those with higher LGI scores had higher WBC (6.8 vs. 10.6 vs. 14.9; *P* < 0.001), CRP (7.6 vs. 8.8 vs. 14.3; *P* < 0.001), NLR (2.8 vs. 8.7 vs. 16.4; *P* < 0.001) and PLT (177.9 vs. 212.4 vs. 246.7; *P* < 0.001) counts.

**Table 1:** Baseline Data According to the tertile of Low-grade inflammation score

		T1 (<=-3) n = 79		T2 (-3 to 2) n = 98	T3 ( > = 3) n = 93		*P*	
***Demographics***								
Age, mean (SD)		59 (9.6)		58 (11.3)	62 (11.2)		0.027	
Male, n (%)		27 (34.2)		33 (33.7)	32 (34.4)		0.994	
***Vascular risk factors***								
Hypertension, n (%)		48 (60.8)		59 (60.2)	65 (69.9)		0.308	
DM, n (%)		7 (8.9)		10 (10.2)	17 (18.3)		0.120	
Current smoking, n (%)		8 (10.1)		16 (16.3)	11 (11.8)		0.438	
**Aneurysmal location, n (%)**							0.580	
Anterior		43 (54.4)		49 (50.0)	55 (59.1)			
Posterior		26 (32.9)		30 (30.6)	26 (28.0)			
Multiple aneurysms		10 (12.7)		19 (19.4)	12 (12.9)			
**Surgical approaches, n (%)**							0.263	
Clipping		3 (3.8)		10 (10.2)	10 (10.8)			
Coiling		75 (94.9)		88 (89.8)	88 (87.1)			
None		1 (1.3)		0 (0.0)	2 (2.2)			
**WFNS grades, n (%)**							< 0.001	
I-III		80 (87.9)		56 (62.2)	39 (43.8)			
IV-V		11 (12.1)		34 (37.8)	50 (56.2)			
**Hunt-Hess grade, n (%)**							< 0.001	
I-III		77 (97.5)		84 (85.7)	59 (63.4)			
IV-V		2 (2.5)		14 (14.3)	34 (36.6)			
**CT Fisher grade, n (%)**							< 0.001	
I-II		69 (87.3)		69 (70.4)	40 (43.0)			
III-IV		10 (12.7)		29 (29.6)	53 (57.0)			
mRS, n (%)							< 0.001	
1–2		7 (8.9)		21 (21.4)	51 (54.8)			
3–6		72 (91.1)		77 (78.6)	42 (45.2)			
SCVS, n (%)		10 (12.7)		23 (23.5)	41 (44.1)		< 0.001	
***Laboratory data***								
WBC count, 10^9^/L, mean (SD)		6.8 (2.3)		10.6 (3.0)	14.9 (4.6)		< 0.001	
CRP, mg/L, median (IQR)		7.6 (3.8–10.9)		8.8 (5.6–13.2)	14.3 (8.9–17.9)		< 0.001	
NLR, median (IQR)		2.8 (1.7–5.9)		8.7 (5.0-14.4)	16.4(12.7–26.3)		< 0.001	
PLT, mean (SD)		177.9 (45.4)		212.4 (64.9)	246.7 (68.6)		< 0.001	
Albumin, g/L, mean (SD)		40.0 (4.2)		41.0 (5.6)	39.7 (5.1)		0.192	

SD, Standard Deviation; IQR, Interquartile Range; P, P for trend; DM, Diabetes Mellitus; mRS, Modified Ranking Scale; WFNS, World Federation of Neurosurgical Societies; WBC, White Blood Cell counts; CRP, C-Reactive Protein; NLR, Neutrophil-to-Lymphocyte ratio; PLT, Platelet

The demographic characteristics, clinical data, and laboratory data of patients with and without SCVS are presented in Tables 2 and 3. Patients with SCVS and poor functional outcomes were older (62 vs. 59; *P* = 0.017) (64 vs. 58; *P* < 0.001); more likely to have a higher CT fisher grade (78.4% vs. 17.3%; *P* < 0.001) (91.1% vs. 10.5%; *P* < 0.01), Hunt-Hess grade (40.5% vs. 10.2%; *P* < 0.001) (57.0% vs. 2.6%; *P* < 0.001) and WFNS grade (73.0% vs. 20.9%; *P* < 0.001) (87.3% vs. 13.6%; *P* < 0.001); and more likely to have higher WBC counts (12.8 vs. 9.8; *P* < 0.001) (14.1 vs. 9.7; *P* < 0.001), CRP levels (16.5 vs. 7.9; *P* < 0.001) (13.2 vs. 7.8; *P* < 0.001), NLR values (13.0 vs. 8.8; *P* = 0.039) (14.7 vs. 7.7; *P* < 0.001) and LGI scores (3.0 vs. -2.0; *P* < 0.001) (4.4 vs. -1.8; *P* < 0.001). However, the albumin level was lower in those patients with SCVS (37.9 vs. 41.1; *P* < 0.001).

**Table 2:** Univariate analysis of association with symptomatic vasospasm

Variables	SCVS (n = 74)	Non-SCVS (n = 196)	*P*
Age, years, mean (SD)	62 (8.5)	59 (11.5)	0.017
Male, n (%)	32 (43.2)	60 (30.6)	0.051
Hypertension, n (%)	52 (70.3)	120 (61.2)	0.168
DM, n (%)	12 (16.2)	22 (11.2)	0.270
Current smoking, n (%)	10 (13.5)	25 (12.8)	0.869
**Aneurysm location, n (%)**			0.859
Anterior	41 (55.4)	106 (54.1)	
Posterior	23 (31.1)	59 (30.1)	
Multiple aneurysms	10 (13.5)	31 (15.8)	
***Surgical approaches, n (%)***			0.247
Clipping	9 (12.2)	14 (7.1)	
Coiling	65 (87.8)	179 (91.3)	
None	0 (0.0)	3 (1.5)	
**CT Fish grade, n (%)**			< 0.001
I-II	16 (21.6)	162 (82.7)	
III-IV	58 (78.4)	34 (17.3)	
**WFNS grade, n (%)**			< 0.001
I-III	20 (27.0)	155 (79.1)	
IV-V	54 (73.0)	41 (20.9)	
***Hunt-Hess grade, n (%)***			< 0.001
I-III	44 (59.5)	176 (89.8)	
IV-V	30 (40.5)	20 (10.2)	
**Rebleeding, n (%)**	6 (8.1)	8 (4.1)	0.183
WBC count, 10^9^/L, median (IQR)	12.8 (8.5–16.0)	9.8 (7.1–12.8)	< 0.001
CRP, mg/L, median (IQR)	16.5 (9.6–21.6)	7.9 (4.6–11.7)	< 0.001
NLR, median (IQR)	13.0 (5.4–19.9)	8.8 (3.3–16.3)	0.039
PLT, mean (SD)	207 (60.4)	217 (69.2)	0.308
LGI score, median, (IQR)	3.0 (0–7)	-2.0 (-5-3)	< 0.001
Albumin, g/L, mean (SD)	37.9 (4.5)	41.1 (5.0)	< 0.001

SD, Standard Deviation; IQR, Interquartile Range; SCVS, Symptomatic Cerebral Vasospasm; WFNS, World Federation of Neurosurgical Societies; WBC, White Blood Cell counts; CRP, C-Reactive Protein; NLR, Neutrophil-to-Lymphocyte ratio; PLT, Platelet; LGI, Low Grade Inflammation

**Table 3:** Univariate analysis of association with functional outcome

Variables	Good outcome (n = 191)	Poor outcome (n = 79)	*P*
Age, years, mean (SD)	58 (10.9)	64 (9.8)	< 0.001
Male, n (%)	59 (30.9)	33 (41.8)	0.086
Hypertension, n (%)	119 (62.3)	53 (67.1)	0.457
DM, n (%)	23 (12.0)	11 (13.9)	0.671
Previous stroke, n (%)	11 (5.8)	14 (17.7)	0.002
Current smoking, n (%)	25 (13.1)	10 (12.7)	0.924
**Aneurysm location, n (%)**			0.676
Anterior	106 (55.5)	41 (51.9)	
Posterior	55 (28.8)	27 (34.2)	
Multiple aneurysms	30 (15.7)	11 (13.9)	
***Surgical approaches, n (%)***			0.453
Clipping	15 (7.9)	8 (10.1)	
Coiling	173 (90.6)	71 (89.9)	
None	3 (1.6)	0 (0.0)	
**CT Fish grade, n (%)**			< 0.001
I-II	171 (89.5)	7 (8.9)	
III-IV	20 (10.5)	72 (91.1)	
**WFNS grade, n (%)**			< 0.001
I-III	165 (86.4)	10 (12.7)	
IV-V	26 (13.6)	69 (87.3)	
***Hunt-Hess grade, n (%)***			< 0.001
I-III	186 (97.4)	34 (43.0)	
IV-V	5 (2.6)	45 (57.0)	
**Rebleeding, n (%)**	8 (4.2)	6 (7.6)	0.251
**SCVS, n (%)**	21 (11.1)	53 (67.1)	< 0.001
WBC count, 10^9^/L, mean (SD)	9.7 (3.9)	14.1 (5.2)	< 0.001
CRP, mg/L, median (IQR)	7.8 (4.6–11.6)	13.2 (9.3–20.0)	< 0.001
NLR, median (IQR)	7.7 (3.1–14.6)	14.7 (7.2–23.2)	< 0.001
PLT, median (IQR)	215 (166.5–255.0)	215 (165.5-255.5)	0.812
LGI score, mean, (SD)	-1.8 (5.0)	4.4 (5.1)	< 0.001
Albumin, g/L, mean (SD)	40.6 (4.9)	39.4 (5.3)	0.072

SD, Standard Deviation; IQR, Interquartile Range; DM, Diabetes Mellitus; SCVS, Symptomatic Cerebral Vasospasm; WBC, White Blood Cell Counts; CRP, C-reactive Protein; NLR, Neutrophil-to-Lymphocyte Ratio; LGI, Low Grade Inflammation

### Association of the LGI score with SCVS and poor functional outcome in multivariate analysis

The results of univariate and multivariate logistical regression are shown in Tables 4 and 5. In the univariate logistical regression, LGI score revealed the ability to predict SCVS and poor functional outcome (OR, 1.186; 95% CI, 1.119–1.256; *P* < 0.001) (OR, 1.267; 95% CI, 1.185–1.355; *P* < 0.001).After adjusting for confounders the LGI score independently predicted SCVS (OR, 1.083; 95% CI, 1.011–1.161; *P* = 0.024) and poor functional outcome (OR, 1.132; 95% CI, 1.023–1.252; *P* = 0.016). Similar significant results were obtained when the LGI was defined as the categorical group, and the risk of SCVS in the second and third tertile group was significantly higher than that in the first tertile group (as the reference value) (OR, 2.826; 95% CI, 1.090–7.327; *P* = 0.033) (OR, 3.243; 95% CI, 1.258–8.358; *P* = 0.015).

**Table 4:** Multivariate logistic regression analyzes the the impact of LGI (as continuous variable) on SCVS and functional outcome

Outcomes		Crude results		Adjusted results	
		OR (95% CI)	*P*	OR (95% CI)	*P*
**SCVS**	**LGI**	1.186 (1.119–1.256)	< 0.001	1.083 (1.011–1.161)	0.024
**Poor outcome**	**LGI**	1.267 (1.185–1.355)	< 0.001	1.132 (1.023–1.252)	0.016

LGI, low grade inflammation; SCVS, symptomatic cerebral vasospasm; OR, odds ratio; CI, confidential interval; P, p for trend;

**Table 5:** Multivariate logistic regression analyzes the the impact of LGI (as categorical variable) on SCVS and functional outcome

Outcomes	LGI	Crude results		Adjusted results	
	distribution	OR (95% CI)	*P*	OR (95% CI)	*P*
**SCVS**	T1	Reference		Reference	
	T2	4.160 (1.924–8.998)	< 0.001	2.826 (1.090–7.327)	0.033
	T3	8.845 (4.081–19.169)	< 0.001	3.243 (1.258–8.358)	0.015
**Poor outcome**	T1	Reference		Reference	
	T2	4.876 (2.211–10.754)	< 0.001	1.296 (0.364–4.610)	0.689
	T3	12.941 (5.829–28.726)	< 0.001	2.275 (0.605–8.548)	0.224

WFNS, World Federation Neurological Societies; LGI, low grade inflammation; OR, odds ratio; CL, confident interval; P, p for trend; SCVS, symptomatic cerebral vasospasm

**Fig. 1:**
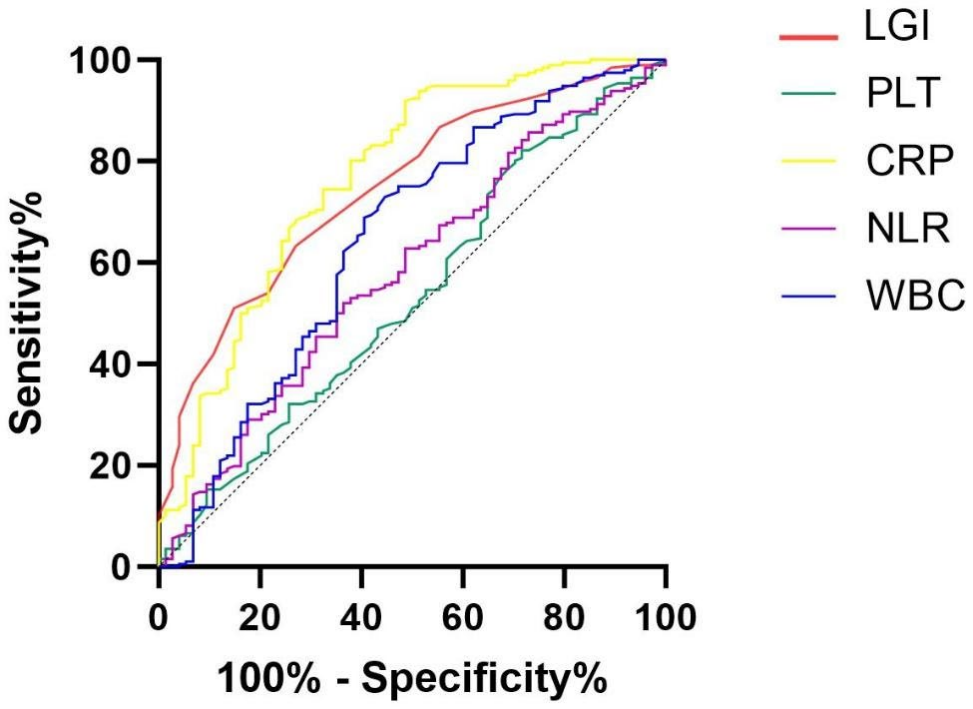
The receiver operating characteristic curves of the low-grade inflammation score and the individual biomarkers to predict SCVS.

**Fig. 2:**
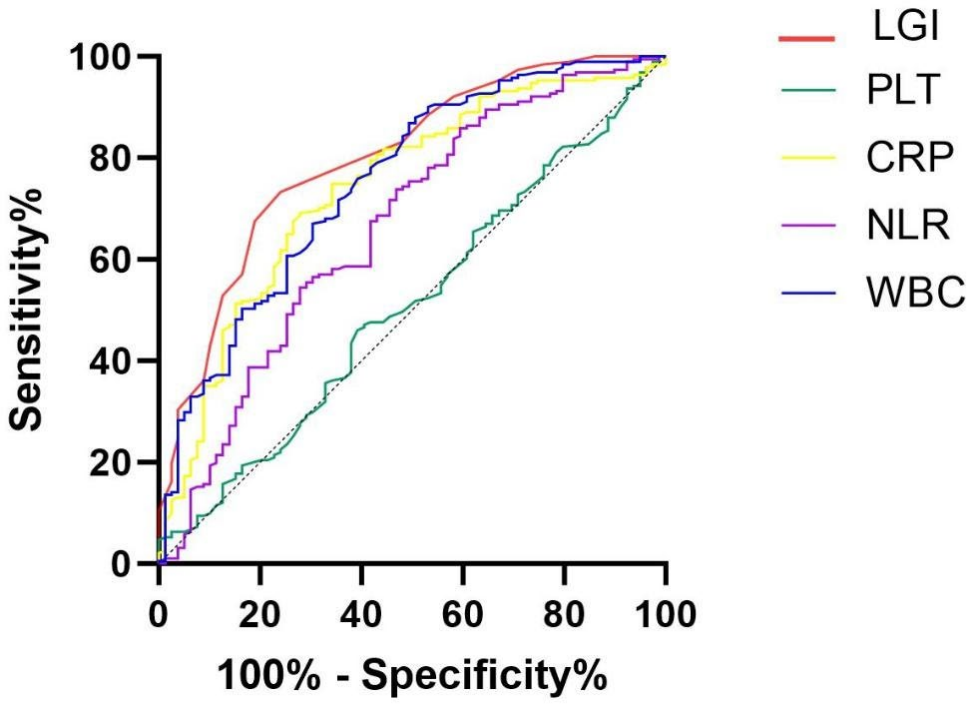
The receiver operating characteristic curves of the low-grade inflammation score and the individual biomarkers to predict poor outcome

Finally, ROC analysis was performed to investigate the ability of the LGI score and individual biomarkers of the score to distinguish between aSAH patients who did or did not develop SCVS and poor functional outcome. The LGI score showed a superior ability to predict SCVS (area under the curve [AUC] = 0.746; 95% CI, 0.690–0.797; *P* < 0.001) and poor functional outcome (area under the curve [AUC] = 0.799; 95% CI, 0.746–0.845; *P* < 0.001). (Figures 1 and 2; Table 6). By pairing and comparing the AUCs of LGI and each individual biomarkers, we found that, the predictive value of LGI on SCVS and poor functional outcome is superior than PLT, NLR and WBC, but there was no statistical difference between LGI and CRP for predicting SCVS (*P* = 0.567) and poor functional outcome (*P* = 0.171) (Table 7**)**.

**Table 6:** ROC curves for SCVS and poor functional outcome

Outcomes		AUC	SE	95% CI	*P* value
SCVS	LGI	0.746	0.0321	0.690–0.797	< 0.001
	CRP	0.770	0.0344	0.715–0.819	< 0.001
	PLT	0.534	0.0402	0.473–0.595	0.382
	NLR	0.581	0.0391	0.505–0.658	0.039
	WBC	0.644	0.0404	0.584–0.701	< 0.001
Poor outcom	LGI	0.799	0.0295	0.746–0.845	< 0.001
	CRP	0.741	0.0335	0.685–0.793	< 0.001
	PLT	0.509	0.0386	0.448–0.570	0.812
	NLR	0.664	0.0380	0.604–0.720	< 0.001
	WBC	0.753	0.0329	0.697–0.803	< 0.001

ROC, Receiver Operating Characteristic; SCVS, Symptomatic Cerebral Vasospasm; AUC, Area Under Curve; CI, Confidential Interval; P, P for Trend; LGI, Low Grade Inflammation Score; CRP, C-reactive Protein; PLT, Platelet; NLR, Neutrophil to Lymphocyte Ratio; WBC, White Blood Cell Counts

**Table 7:** The results of Delong test

Outcomes		SE	95% CI	Z value	*P* value
SCVS	LGI vs. CRP	0.0417	-0.058-0.106	0.573	0.567
	LGI vs. PLT	0.0603	0.094–0.330	3.510	< 0.001
	LGI vs. NLR	0.0345	0.098–0.233	4.789	< 0.001
	LGI vs. WBC	0.0341	0.035–0.169	2.990	0.003
Poor outcome	LGI vs. CRP	0.0418	-0.025-0.139	1.370	0.171
	LGI vs. PLT	0.0388	0.213–0.366	7.454	< 0.001
	LGI vs. NLR	0.0317	0.072–0.197	4.242	< 0.001
	LGI vs. WBC	0.0302	-0.014-0.105	1.505	0.132

SCVS, Symptomatic Cerebral Vasospasm; SE, Standard Error; CI, Confidential Interval; P, P for Trend; LGI, Low Grade Inflammation Score; CRP, C-reactive Protein; PLT, Platelet; NLR, Neutrophil to Lymphocyte Ratio; WBC, White Blood Cell Counts

## Discussion

In this study, we used plasma (CRP) and cellular (WBC count, PLT count and NLR) values to construct an LGI score in aSAH patients. The results of this study indicated that the LGI score was independently associated with SCVS and poor functional outcome at 3 months in aSAH patients. Moreover, the discriminatory ability of the LGI score for poor functional outcomes is superior to that of some individual biomarker.

The LGI score is a composite score that is used to evaluate comprehensive effects on stroke recurrence and total mortality. An increased LGI score is significantly associated with a higher incidence of stroke recurrence and total mortality [12, 13]. Although the possible mechanisms are not well defined, obviously, the inflammatory pathway is the common denominator involving pathogenetic mechanisms among several diseases.

Previous studies have shown that aSAH patients have a dramatic elevation of sympathetic nervous activity, and sympathoexcitation contributes to the elevation of systemic levels of catecholamines [17], inflammatory cytokines and cells [18]. Experimental evidence has shown that the cerebrovasculature displays a super sensitivity to catecholamines after SAH [19], and the spasmogenic ability of these amines may be involved in the genesis of cerebral vasospasm. In addition, sympathetic nervous system overactivation may contribute to cardiac disturbance and marked blood pressure elevation [20], and the instability of physical conditions and acute stress may be appropriate explanations for poor functional outcomes. Since the sympathetic nervous system plays a key role in regulating the inflammatory process [21], it seems reasonable to use inflammatory factors to assess the level of sympathetic activation in clinical practice.

The early-phase proinflammatory cytokine cascade has been postulated to play a crucial and unifying role in the pathogenesis of cerebral vasospasms and poor functional outcome. Subarachnoid blood is a stimulant that induces the transcription of multiple components of the inflammatory cascade [22]. The main manifestations of the neuroimmune system in aSAH patients are excessive neuroinflammation and immunodepression, which can be indirectly indicated by neutrophil increases and lymphocyte decreases [23, 24]. Excessive accumulation of neutrophils in the central nervous system is involved in early brain injury, and lymphocyte depletion after aSAH may lead to some adverse complications, which are potential mechanisms of poor prognosis in aSAH patients [25]. NLR is a novel marker of systemic inflammatory response, and the peripheral NLR may reflect the severity of neutrophil infiltration after aSAH. Studies have shown that NLR is an independent predictor of poor outcome and DCI occurrence in aSAH [5]. In addition, systemic leukocytosis is commonly observed in SAH patients, and white blood cells can directly promote free radical formation and release cytokines and chemotactic factors to propagate immune dysregulation [26]. WBC infiltration and neutrophil recruitment all contribute to SCVS by weakening microvascular perfusion and leading to the release of a large number of inflammatory mediators [27]. These characteristics make the WBC count a reliable index to predict DCI after aSAH [28, 29]. Platelet activation and aggregation are also involved in the pathogenesis of DCI, and the potential role of platelets in microthrombi formation, large artery vasospasm, microvessel construction, inflammation and cortical spreading depolarization may all contribute to the pathophysiology of DCI. CRP is an exquisitely sensitive systemic marker of inflammation and tissue damage [30]. In the clinic, CRP has good prognostic value for aSAH [31] and several other diseases. However, CRP is a nonspecific inflammation biomarker that can be elevated in the presence of any tissue [32]. In clinical practice, measurement of CRP is often combined with that of peripheral inflammatory cells to improve its clinical predictive value. CRP and several peripheral inflammatory cells are routinely collected from aSAH patients and are potentially simple ways for clinicians to determine the risk of SCVS and poor prognosis in aSAH patients.

Each individual inflammatory biomarker of the LGI score is involved in the pathophysiology of aSAH through different inflammatory pathways. However, compared to the role of a single inflammatory biomarker, the LGI score accounts for the possible synergistic effect of each biomarker, effectively controlling the variability of the inflammatory biomarkers. In this study, the predictive effect of the LGI score was superior to that of each individual biomarker, and it was a better predictor of poor outcome and SCVS in aSAH patients.

To our knowledge, this is the first study to use a composite score of several biomarkers to assess the risk of SCVS and poor prognosis in aSAH patients. The present study has several potential limitations that should be addressed when interpreting the results. First, this is a retrospective study in which we excluded patients with incomplete data, which inevitably produced bias. Second, the sample size was quite small, and the study was performed in a single country, which might limit the generalizability of the results to other patient cohorts. Third, we only evaluated the prognosis of patients at 3 months after discharge; thus, long-term follow-up data are needed to support the findings of this study. Fourth, in this study, only the admission LGI score was measured, and other time points of the LGI score were not considered. Finally, some other variables related to outcome (such as intracranial hypertension) were not included in the multivariate logistical regression.

In conclusion, an increased LGI score can be a useful predictor of SCVS and poor functional outcome after aSAH, and the predictive value of the LGI score for poor prognosis is better than that of each individual inflammatory biomarker.

## Data Availability

The datasets analyzed during the current study are available from the corresponding author on reasonable request.
